# Understanding and Designing the Strategies for the Microbe-Mediated Remediation of Environmental Contaminants Using Omics Approaches

**DOI:** 10.3389/fmicb.2018.01132

**Published:** 2018-06-04

**Authors:** Muneer A. Malla, Anamika Dubey, Shweta Yadav, Ashwani Kumar, Abeer Hashem, Elsayed Fathi Abd_Allah

**Affiliations:** ^1^Department of Zoology, Dr. Harisingh Gour University, Sagar, India; ^2^Metagenomics and Secretomics Research Laboratory, Department of Botany, Dr. Harisingh Gour University, Sagar, India; ^3^Department of Botany and Microbiology, College of Science, King Saud University, Riyadh, Saudi Arabia; ^4^Department of Plant Production, College of Food and Agricultural Sciences, King Saud University, Riyadh, Saudi Arabia

**Keywords:** environmental pollution, metagenomics, metatranscriptomics, proteomics, metabolomics, fluxomics, bioremediation

## Abstract

Rapid industrialization and population explosion has resulted in the generation and dumping of various contaminants into the environment. These harmful compounds deteriorate the human health as well as the surrounding environments. Current research aims to harness and enhance the natural ability of different microbes to metabolize these toxic compounds. Microbial-mediated bioremediation offers great potential to reinstate the contaminated environments in an ecologically acceptable approach. However, the lack of the knowledge regarding the factors controlling and regulating the growth, metabolism, and dynamics of diverse microbial communities in the contaminated environments often limits its execution. In recent years the importance of advanced tools such as genomics, proteomics, transcriptomics, metabolomics, and fluxomics has increased to design the strategies to treat these contaminants in ecofriendly manner. Previously researchers has largely focused on the environmental remediation using single omics-approach, however the present review specifically addresses the integrative role of the multi-omics approaches in microbial-mediated bioremediation. Additionally, we discussed how the multi-omics approaches help to comprehend and explore the structural and functional aspects of the microbial consortia in response to the different environmental pollutants and presented some success stories by using these approaches.

## Introduction

**Figure 1:**
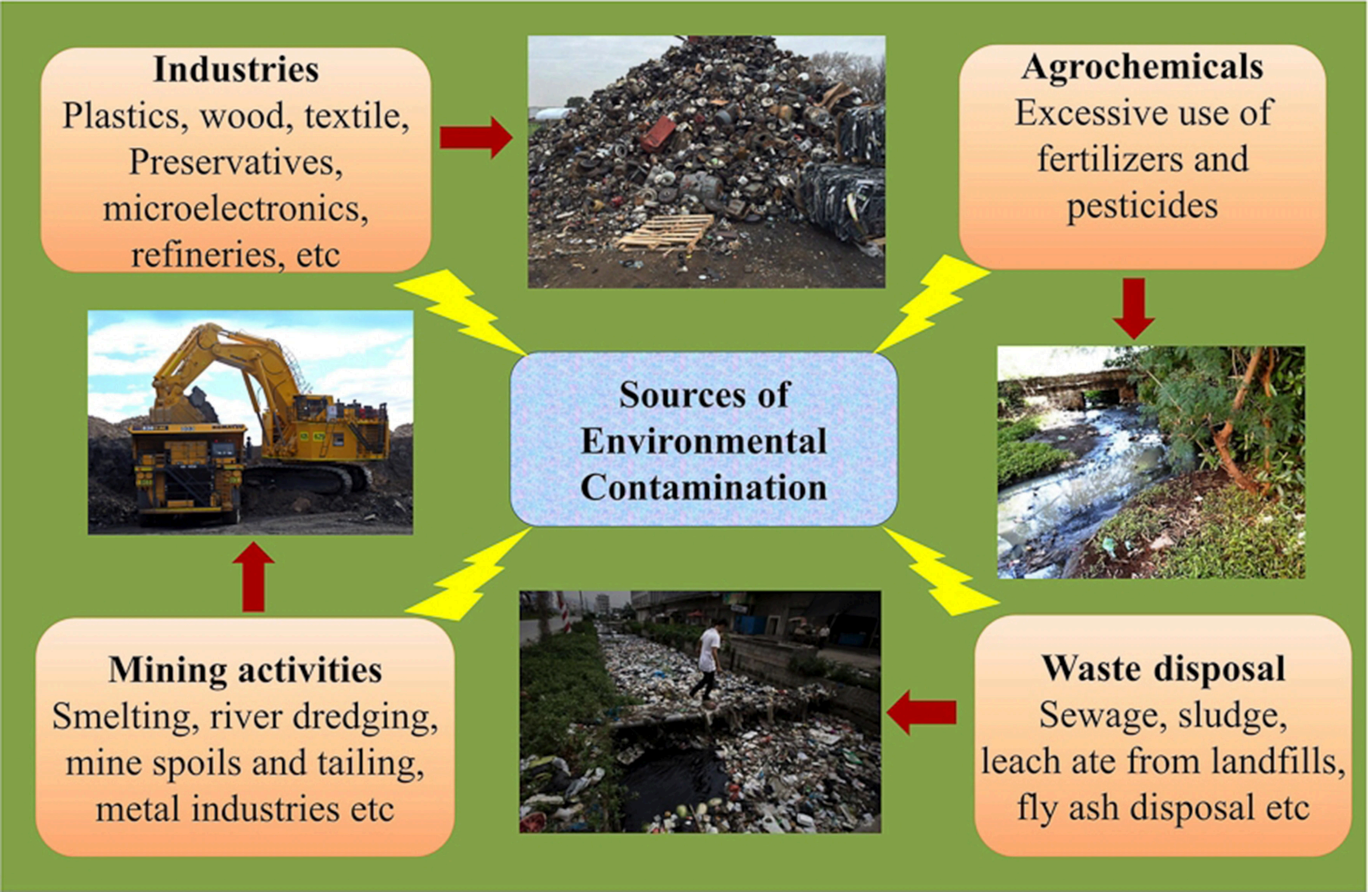
Different sources of environmental contamination.

The quality of life on Earth is inevitably related to the overall quality of the environment. As human activity has increased around the globe, the Earth has been contaminated with a large number of toxic pollutants from multiple sources (Raghunandan et al., 2014, 2018). The shrinking of natural resources, an increase in pollution and carbon emissions and other problems related to human health are the consequence of industrialization and have proven disastrous for every global region (Ahuti, 2015). Industrialization not only entails hi-tech innovations but also affects the economic and social transformation of human societies (Mgbemene et al., 2016). The industrial revolution, has resulted into hazardous health problems that are amplified by the large-scale environmental contaminations (Figure 1). The advancements in the field of technology and industrialization, brings with them, their obnoxious partners, pollution as well as degradation of the environment. These revolutions have led to both intentional and accidental discharges of toxic gases, chemicals, and xenobiotics into the environment.

Environmental contamination is a pertinacious problem and continues to be a burden to human health. While a number of approaches have been used to monitor and reduce this problem, it remains a difficult issue. Globally, both the environment and humans are affected by these hazards. To safeguard both humans and environment from the adverse consequences of environmental pollution novel approaches must be designed, and bioremediation is one such approach. Interest in the microbial-based bioremediation of contaminants has increased in recent years, as people endeavor to find sustainable ways of remediating polluted environments (Raghunandan et al., 2014, 2018; Kumar et al., 2016). The biotransformation and bioremediation-based methods strive to harness the naturally occurring microbial catabolic diversity to degrade, transform or accumulate vast amounts of problematic compounds, including radionuclides, metals, pharmaceutical substances, polyaromatic hydrocarbons (PAHs), and polychlorinated biphenyls (PCBs). Advancements in NGS (next generation sequencing) in recent years has allowed detailed genomic, metagenomic, and bioinformatic analyses of environmentally important microorganisms, thereby providing unprecedented insights into key biodegradative pathways. Mukherjee et al. (2017), studied the microbial responses to hydrocarbon-contaminated environments and suggested that substantial taxonomic and functional variation occurs in different geographically and spatially isolated oil-polluted sites. In addition to providing significant analytical and visual methods for understanding the relation between the soil microbiome and ecosystem functioning, their study provided novel insights into the ecological dynamics of hydrocarbon-contaminated sites. A study carried out by Luo et al. (2014) revealed that soil microbial diversity varies in response to heavy metal contamination. Hauptmann et al. (2017) investigated microbial metagenomes from the ice sheets of Greenland and isolated potential microbial genes for the degradation and resistance to contaminants, such as heavy metals (lead and mercury), polycyclic aromatic hydrocarbons (PAHs), and polychlorinated biphenyls (PCBs). Similar studies were carried out by Joshi et al. (2014) that investigated the metagenomes isolated from petroleum muck, which revealed the indigenous microbial communities inhabiting the petroleum-contaminated sites. Recently, a number of research papers (George et al., 2011; Kumavath and Deverapalli, 2013; Bell et al., 2014; Pushpanathan et al., 2014; Ufarté et al., 2015; Czaplicki and Gunsch, 2016; Chistoserdova, 2017) have been published wherein different authors have emphasized individual genomic or transcriptomic approaches in bioremediation of environment. Previously researchers has largely focused on the environmental remediation using single omics-approach, however the present review specifically addresses the integrative role of the multi-omics approaches in microbial-mediated bioremediation. Additionally, we discussed how the multi-omics approaches help to comprehend and explore the structural and functional aspects of the microbial consortia in response to the different environmental pollutants and mentioned some success stories by using these approaches.

## Mechanism of the bioremediation process

**Figure 2:**
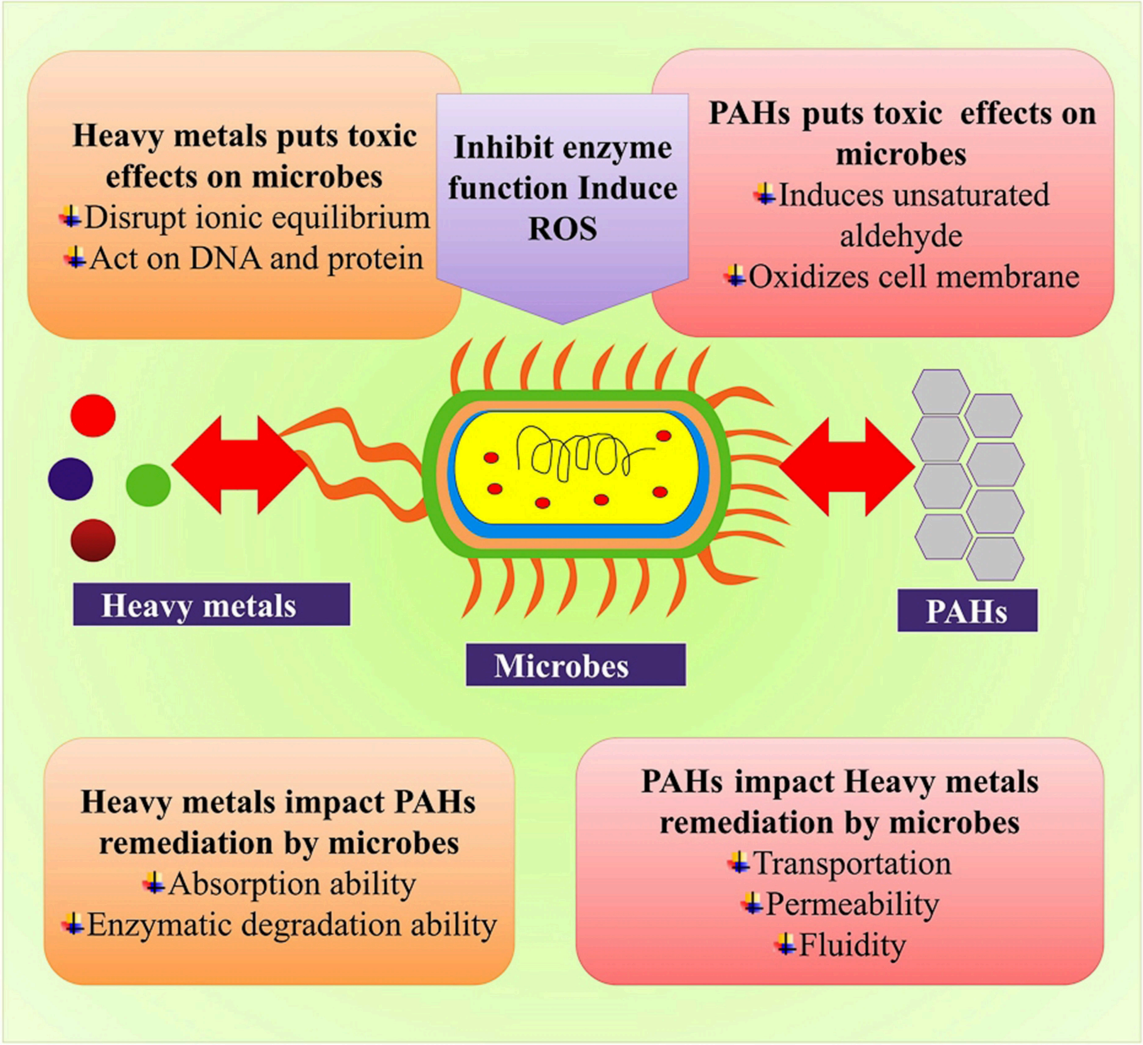
Microbial-based bioremediation mechanism.

Bioremediation is the application of microbes to degrade environmental pollution. It is an ecologically sound and state of art practice that utilizes microbial processes to completely remove the toxic contaminants. Microorganisms are not only important in regulating the biogeochemical cycles (Griggs et al., 2013), perpetuating the atmosphere (Morris et al., 2011), keeping us healthy and suppressing the plant diseases and helping them to grow (Pineda et al., 2017) but also play their part in cleaning of environmental pollutants (Morris et al., 2011). Microbial mediated bioremediation is of great significance because it promises a cheaper, simpler and more environmentally friendly method when compared to the more commonly employed “muck, suck and truck” non-biological remedial methods, in which the contaminants are simply pumped up or dug out and are then shipped elsewhere (Lovley, 2003). However, the promise of bioremediation is yet to be fully realized. One reason for this is that bioremediation approaches that are successful at one location may not be effective in other locations. Additionally, the microbial processes that remediate pollutants under lab conditions may fail to perform adequately in the field. The reasons for such failures are, however, unclear, and as a result many managers are unwilling to use bioremediation as an option for environmental clean-up. Moreover, the mechanisms that control the growth and activity of microorganisms in contaminated environments are not well understood, thereby limiting the implementation of bioremediation (Lovley, 2003). Dynamic behavior, nutritional flexibility and knack of adapting to extreme environmental conditions make microbes the most suitable life forms for endurance. This feature of microbes is advantageous and beneficial to the mankind particularly when it comes to elimination of pollutants and other toxic compounds from the environment. Microbes have the tendency to degrade contaminants from the environments and do so via diverse enzymatic process, thus mitigating or removing the environmental contaminations (Lovley et al., 1991). An extensive list of the microbes that carry out the bioremediation processes is available (Satyanarayana et al., 2012; Prakash et al., 2013; Abou Seeda et al., 2017). Microorganisms can carry out environmental restoration through a diverse array of processes, such as binding, oxidation, volatilization, and immobilization or by chemical transformation of the pollutants. One of the most common types of the bioremediation technique is the oxidation of the toxic organic pollutants to the harmless products (Figure 2). Oxygen the most common electron acceptor for microbial respiration as well as the agent for the aerobic degradation of wide range of organic pollutants ranging from arenes such as from benzene to xenobiotics (pesticides) has been studied in detail (Wackett and Hershberger, 2001). While a vast phylogenetic diversity is able to degrade aerobic pollutants (Wackett and Hershberger, 2001), but *Pseudomonas species* and its close associates are the most intensively investigated organisms due to their ability to degrade many different contaminants.

Ideally, bioremediation approaches should be designed based on knowledge of the particular microorganisms inhabiting the contaminated areas, including their metabolic processes and how the microorganisms react to changes in the environmental conditions. Unfortunately, in practice, this specialized knowledge is not easily available, and the use of microbes in bioremediation is extremely experimental rather than knowledge-based. Although at present the science of bioremediation is still far from ideal, it now seems achievable. Common terminologies used in this article are given in Table S1.

## Strategies to study microbial mechanisms with an aim to expedite the bioremediation process

### Pre-genomics access to bioremediation: culture-based techniques

Microbes are the most diverse and profuse forms of life on earth, and they emerge as key players in important ecological processes, such as organic matter putrefaction, soil structure formation, and the recycling of important chemical elements. Thus, microbes play a vital role in regulating the global biogeochemical cycles (Garbeva et al., 2004). Knowledge regarding microbial dynamics and their interactions with biotic and abiotic factors is an indispensable tool in the fields of bioremediation, biotechnology, pharmacy and energy production processes. Currently, the majority of studies that are related to bioremediation processes rely on the “treatability study,” in which samples from contaminated sites are typically incubated under laboratory conditions, and the rates at which the contaminants are immobilized or degraded are recorded (Head et al., 2003). These studies provide an estimate regarding the potential metabolic activities of the microbial consortia but give little insight into the microbes responsible for bioremediation. When bioremediation processes are studied more precisely, an effort is made to isolate and characterize the organisms that are responsible for remediation (Head et al., 2003). The major drawback of culture-dependent techniques is that greater than 99% of microbes that inhabit the diverse natural environments are either uncultivable or are very difficult to culture (Vartoukian et al., 2010; Dickson et al., 2014; Bursle and Robson, 2016). The recovery of microbial isolates that are responsible for bioremediation processes is invaluable because the study of these isolates provides an opportunity to scrutinize their biodegradation reactions, along with other physiological aspects that are liable to control growth and other activities in the polluted environments. To overcome these limitations and drawbacks, a number of DNA-based molecular techniques have been devised to explore the microorganisms responsible for bioremediation.

### Molecular approaches to study microbial bioremediation

For years microbiologists have sought a reliable technique to ascertain the microbial diversity in environmental samples. A number of molecular methods have been developed for this purpose. The development of the analysis of 16S rRNA gene sequences has significantly enhanced our ability to understand and explore the microbial world (Armougom and Raoult, 2009; Ju and Zhang, 2015). This has countered the idea that only a small percentage of the bacteria are isolated using culturing methods (Carroll and Patel, 2015). Denaturing gradient gel electrophoresis is applied in the field of microbial ecology to profile the complex microbial diversity and is devoid of the biases inherent in culture analysis (Fakruddin and Mannan, 2013). Molecular-based approaches such as denaturing gradient gel electrophoresis allows the separation of 16S rRNA genes based on the decreased electrophoretic mobility of partially melted double stranded DNA (dsDNA) in polyacrylamide gel that contains either a linear gradient of temperature or a linear gradient of DNA denaturant (a mixture of formamide and urea). The primary advantage of this technique is that it generates a profile of the entire diversity of the microbial community by separating a mixed population of 16S rRNA gene products. However, these techniques have several limitations, biases, and drawbacks. For example, the dominant populations are better revealed, and the bands obtained from multiple numbers of species may be obscured behind a single band, thereby leading to an underestimation of the microbial diversity (Satokari et al., 2001; Gafan and Spratt, 2005; Green et al., 2010). Recently, DGGE based analysis of 16S rRNA sequences has been used to investigate and profile complex microbial diversity and to deduce the phylogenetic affiliation among these microbial communities (Nishimura et al., 2010).

## Role of omics-approaches in microbial bioremediation

Microbial-bioremediation process utilizes the indigenous microbial communities to clean up the environmental contaminations. The rate at which the contaminants are detoxified depends on a number of factors such as the composition of the indigenous microbial communities, nature, and extent of the pollutant and environmental conditions (Chakraborty et al., 2012). Thus, the optimization of the bioremediation process demands the combination of various complex variables, to understand and envisage the fate of environmental pollutants. Molecular approaches such as genomics, proteomics, transcriptomics, metabolomics, and fluxomics are now consistently finding their applications in bioremediation process so as to understand the exact mechanism involved. the advent of NGS methods and *in silico* analyses have enabled the environmental microbiologists to address these problems and has helped them to open up the microbial “balckbox” in contaminated environments (Maphosa et al., 2010). A number of microbes haves been reported that carry out the degradation of different environmental contaminants (Table 1). The application of omic-tools for the taxonomic and functional aspects of the microbial communities from contaminated sites has led to the discovery of some novel bacteria that otherwise were not accessible by using the traditional culturing techniques. Recently, high through-put omics-approaches have been employed to explore the systems biology of the microbial consortia in myriad of environments. However, the successful implementations of these multifaceted bioremediational approaches necessities a much detailed and comprehensive understanding of the factors that govern the growth, metabolism, structure, dynamics, and functions of the indigenous microbial consortia of these sites. Here is where the recent advances and breakthroughs in genomics, metatranscriptomics, metaproteomics metabolomics, and fluxomics along with *in silico* (bioinformatics) analysis play their part by providing key in-sights in understanding and exploring the microbial communities and their mechanisms in the bioremediation of environmental contaminants. These approaches have made it practically possible and economically feasible to explore the metagenomes of contaminated environmental samples, harboring diverse microbial communities. This has not only provided an insight regarding the diversity, but also putative information about the meta-functionality of the microbial populations inhabiting the contaminated environments. Even the combination of data generated via different omic-approaches may be used to study the microbial metabolism during the bioremediation processes. Studies like these will provide an opportunity to develop efficient strains of microbes, so as to improve metabolism of different xenobiotics (Desai et al., 2010). The efficiency of the bioremediation will definitely be increased if the precise molecular approaches are properly used and scientifically pursued.

**Table 1:** Different types of microbes used in the bioremediation of various environmental contaminants.

**Microorganism**	**Types of pollutant degraded**	**Significant outcomes**	**References**
*Brevibacterium epidermidis* EZ-K02	Industrial wastes	*Brevibacterium epidermidis*, a bacterium that is capable of degrading waste waters contaminated with large scale dissolution of chemical compounds and nitrocellulose particles	Ziganshina et al., 2018
*Bacillus* sp. CDB3 *Lysinibacillus sphaericus*	Arsenic	*Bacillus* sp. CDB3, *Lysinibacillus sphaericus* Shows high resistance to arsenic contamination and aids in treating arsenic poisoning.	Rahman et al., 2016 Yang and Zhang, 2017
*Mycobacterium dioxanotrophicus*	Heterocyclic organic compounds (Dioxane)	*Mycobacterium dioxanotrophicus* is capable of remediating various heterocyclic organic compound contaminated environments by making the use of 1, 4-dioxane as a single source of energy and carbon and energy	He et al., 2017
*Hyphomicrobium* sp. Strain GJ21	Dichloromethane	*Hyphomicrobium* degrades halogenated contaminants by making the use of dichloromethane as a source of both energy and carbon	Bringel et al., 2017
*Microbacterium oleivorans* Strain A9	Radionuclides	*Microbacterium oleivorans* strain A9, a radionuclide-resistant actinobacterium capable of degrading uranium	Ortet et al., 2017
*Plantibacter flavus* Strain 251	Hydrocarbon-contaminated environments	*Plantibacter flavus* isolate 251 is known to possess novel biodegradation enzymes. The bacterium is anticipated to provide some of the novel insights into exploiting the hydrocarbon degrading pathways	Lumactud et al., 2017
*Bacillus subtilis* SR1	Polyaromatic hydrocarbon	*Bacillus subtilis* SR1 is a bacterium besides showing resistance to heavy-metals is also capable of degrading polyaromatic hydrocarbons	Kotoky et al., 2017
*Alkaliphilus metalliredigens* Strain QYMF,	Heavy metals	*A* metal-reducing bacterium capable of thriving in alkaline environments, a feature that is not commonly found in metal respiring microbes	Hwang et al., 2016
*Pseudomonas veronii* Strain 1YdBTEX2	Aromatic solvents *viz.*, Benzene, toluene, ethyl benzene, Xylene (BTEX)	*Pseudomonas veronii* bacterium contains genes that carry out the degradation of aromatic solvents via a catabolic pathway	Junca and Pieper, 2004 Morales et al., 2016 Moreno-Forero et al., 2016
*Pseudomonas plecoglossicida* TND35	Nicotine	*Pseudomonas plecoglossicida* TND35 besides being an effective nicotine-degrading bacterium also has genes responsible for the degradation of heavy metals, aromatic compounds, and biosynthesis of butanol	Raman et al., 2015
*Microbacterium* spp.,	Heavy metals	*Microbacterium* spp plays an important role in phytoextraction and mobilization of heavy metals	Corretto et al., 2015
*Arthrobacter* sp. Strain SPG23	Hydrocarbon degradation	*Arthrobacter* is a hydrocarbonoclastic Gram-positive bacterium and is a potent bacterium for used for the remediation of the diesels fuels	Gkorezis et al., 2015
*Raoultella ornithinolytica-*TNT	Trinitrotoluene	*Raoultella ornithinolytica-*TNT is a Gram-negative bacterium. Strains of TNT make use of nitrate released from trinitrotoluene thereby making it less toxic. Hence is considered as a potent microbe in terms of bioremediation applications.	Thijs et al., 2014
*Pseudomonas taeanensis*	Hydrocarbons (Petroleum compounds)	*Pseudomonas taeanensis* a bacterium that is able to degrade petroleum compounds like diesel, kerosene and gasoline	Lee et al., 2014
*Caulobacter* sp. Strain OR37	Heavy metals	Possesses tolerance to elevated concentrations of heavy metals *viz.*, cadmium, cobalt, uranium, nickel	Utturkar et al., 2013
*Ochrobactrum pseudogrignonense*	Arsenic pollutants	*Ochrobactrum pseudogrignonense*, a highly potent and efficient arsenate-resistant bacterium. The bacterium is involved in the degradation of the arsenic from arsenate-contaminated soils	Yang et al., 2013
*Arthrobacter* sp. Strain SJCon	2-Chloro-4-Nitrophenol	*Arthrobacter* sp. strains are useful in drafting the genetic pathways that are involved in the bioremediation of the aromatic compounds	Vikram et al., 2013
*Brachybacterium sp*. *Cytophaga sp*. *Sphingomonas sp*. *Pseudomonas sp*.	Oil spills	The bacteria are capable of remediating the oil-contaminated sites *via*, the processes of biostimulation and bioaugmentation	Angelim et al., 2013
*Pseudomonas aeruginosa*	Organic and Inorganic mercury	*Pseudomonas aeruginosa* bacterium is one of the potent agent that carries out the bioremediation of both organic and inorganic mercury in highly contaminated mercury sites	Dash and Das, 2012
*Moraxella saccharolytica* *Alteromonas Putrefaciens* *Klebsiella pneumonia* *Pseudomonas fragi*	Diesel hydrocarbon	*Moraxella saccharolytica, Alteromonas Putrefaciens, Klebsiella pneumonia, Pseudomonas fragi*, a complete bacterial consortium is proved to be one of the better and reliable choices for the rapid and complete remediation of the diesel hydrocarbon contaminated environments	Sharma and Rehman, 2009
Sphingomonadaceae/Sphingomonas	Degrades Hexachlorocyclohexae	Sphingomonads offer biostimulation of HCH polluted sites *via* addition of nutrients and aeration.	Dadhwal et al., 2009
*Pseudomonas putida* strains *P. putida* G7 *P.aeruginosa* PaK1 *P.putida* BS202 *P*. sp. strain U2 *Rhodococcus sp*. NCIMB12038 *Pseudomonas putida* OUS82 *Alcaligenes faecalis* AKF2 *Nocardiodes sp*. KP7	Degradation of PAHs Naphthalene and Phenanthrene	Isolation of the bacterial strains such as *Pseudomonas putida, P. putida* G7, *P.aeruginosa* PaK1, *P. putida* BS202, *P*. sp. strain U2, *Rhodococcus sp*. NCIMB12038 and *Pseudomonas* putida OUS82, *Alcaligenes faecalis* AKF2, *Nocardiodes sp*. KP7via the development of the modern genetic technologies provided a major breakthrough in the PAH remediation. The bacteria are capable of degrading substrates like phenanthrene, Pyrene and benzo-pyrene, fluoranthene, Naphthalene	Chauhan et al., 2008
*Mycobacterium sp*. PYR-1	Degradation of PAHs Pyrene	The strains of the *Mycobacterium* sp. although not producing the biosurfactants possess a strong ability to degrade even the low concentrations of aqueous-phase anthracene	Chauhan et al., 2008
*Neisseria elongate Acinetobacter faecalis* *Staphylococcus. sp*.	Crude petroleum oil	*Neisseria elongate, Acinetobacter faecalis, Staphylococcus. sp.*, are the potent bacterial isolates and carry upto 93, 94, and 95% of hydrocarbon degradation, respectively	Mukred et al., 2008

**Figure 3:**
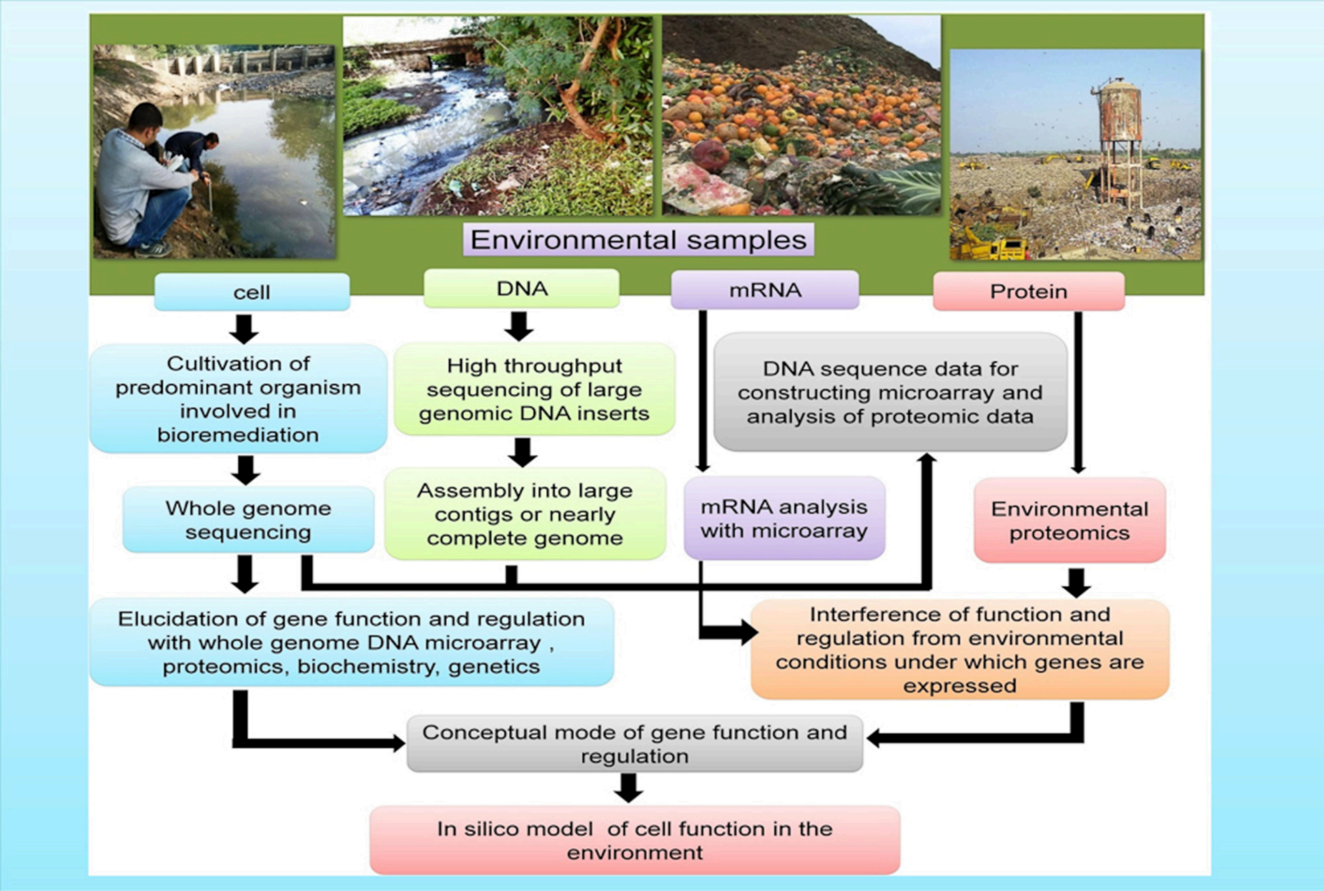
Genome-based approaches that contribute toward the development of models of how microbes function in the polluted environments (a) Cells: the isolated cells from the environmental samples gives an opportunity to furnish/obtain information on the gene composition as well as in-depth physiological analysis (b) DNA: Genomic DNA extracted from the environmental samples provides information and knowledge regarding the genetic potential of the yet-unculturable microorganisms (c) mRNA and Proteins: mRNA and proteins extracted from the environments furnishes information about the gene expression under varied environmental conditions.

Combined results from various “omics tools” has offered key insights regarding the survival, metabolism, and interaction of the microorganisms in their native environments including gut microbiomes (Gill et al., 2006), deep-sea sediments (Hu et al., 2010), groundwater and marine systems (DeLong, 2005; Benndorf et al., 2007; Hemme et al., 2010), and extreme milieus (Baker and Banfield, 2003). In order to expedite the complete remediation of contaminated environments a comprehensive understanding of the physiology, biochemistry, ecology, and phylogeny of the indigenous microbial consortia of contaminated sites is warranted. The application of genome-based techniques in the investigation of both environmental samples and pure cultures makes it possible to build the models that are required to predict the activity of microbes under diverse bioremediation strategies (Figure 3).

### Culture-independent insight: metagenomics

The field of metagenomics is undergoing a rapid evolution amid the arrival of next generation sequencing technologies. The science of metagenomics has bypassed the need for cloning and has facilitated a new approach of comparative metagenomics. Advances in high-throughput technology have revolutionized the field of microbiology. Metagenomics, a rapidly growing and young field of research, aims to investigate uncultured organisms in order to understand the true diversity of microbes, their functions, cooperation, interactions, and evolution within diverse environments. A relatively new approach of molecular biology, metagenomics was used for the first time by Handelsman et al. (1998) while studying the chemistry of unknown soil microbes. Metagenomics involves a culture-independent sequencing-based analysis of DNA that is isolated from the environmental samples, i.e., metagenomes (Daniel, 2005). Sequence phylotyping provides reliable information regarding both diversity (What microorganisms are there?) and function (What can the microorganisms do?). Metagenomics has helped us in closing the gap left by culturing techniques and has provided insights into *in situ* microbial structures, dynamics and functioning thereby enhancing bioremediation processes.

## Approaches to metagenomic analysis

Metagenomic approaches generally fall into one of two categories: function based or sequence-based. Together, these approaches have increased our understanding of the unculturable microbial world and have, therefore, also provided insights into the prokaryotic world that is otherwise obscure.

### Sequence-based analysis

Sequence-based metagenomic analysis provides microbial information irrespective of culturing. In comparison to functional screening, the sequence-based approach depends on the sequence analysis to provide a basis for function prediction. Substantial databases are catalogd in the “Environmental Genome Sequence” database, and the sequencing assignments become more instructive and informative over time because data are continuously compiled from diverse sources. Sequence-based metagenomic analysis can be used for gene identification, genome assemblages, clarifying complete metabolic pathways and comparing organisms from different communities.

### Function-based analysis

Functional metagenomics is a potent and powerful method for studying the functional aspects of genes. Function-based metagenomic analysis involves isolating DNA from environmental samples to study the functions of the encoded proteins (Lam et al., 2015). In functional-based metagenomic analysis, DNA fragments are cloned, expressed in a laboratory host and screened for enzymatic activities. The function-based metagenomic approach allows the discovery of novel genes, and metagenomic sequencing offers unique opportunities to explore novel environments that have yet to be studied. Functional analysis plays a pivotal role in these studies by giving important information regarding the metabolic and functional diversity (Prakash and Taylor, 2012).

## Metagenomics in bioremediation

Environments where anthropogenic activities are widespread have often been contaminated by various types of toxic compounds (Pacwa-Płociniczak et al., 2011). This contamination varies and affects the most important aspects of our ecosystem, such as the air, water, and soil (Saharan et al., 2012). The relationship between species diversity and ecosystem function has long been an area of interest (Sutherland et al., 2013). Disturbances caused by anthropogenic activities, such as contamination by hydrocarbons (van Dorst et al., 2014) and heavy metals (Pessoa-Filho et al., 2015), can strongly affect the microbial diversity and structural composition. Next generation sequencing provides an opportunity for extensive analysis of environmental genomes. Metagenomics, along with other molecular techniques, has revolutionized the field of microbiology by focusing on microbial diversity, evolution and adaption (Riesenfeld et al., 2004). Studies that have investigated the microbial communities from diverse environments, such as sediments and marine water (DeLong et al., 2006; Yooseph et al., 2007), the human gut (Turnbaugh et al., 2007), soils (Smets and Barkay, 2005), and acid mine drainage (Tyson et al., 2005), have generated novel insights into the metabolism, community structure, evolution, function, and genetic makeup of these communities. Metagenomic analysis presents an exceptional opportunity to comprehensively analyse the response of an ecosystem to environmental changes; however, as yet, there are no reported studies that have examined the adaptation and response of microbial communities to environmental pollutants. Metagenomics holds great promise for the field of bioremediation, as it will help to shape the approach to bioremediation in a number of interconnected ways (Satyanarayana et al., 2012; Tripathi et al., 2018). Metagenomic bioremediation offers more positive results with better degradation ratios when compared to other approaches to bioremediation (Kosaric, 2001). First, metagenomics has greatly increased our understanding of how microbes develop “bucket-brigades” for the degradation of xenobiotic compounds, thereby allowing the differentiation of contaminated sites into areas where the native microbiota is able to remediate the environmental status by using intensive *ex situ* treatment or by *in situ* bioaugmentation. Second, it will help to identify key microbial processes and will specify how the community composition could best be complemented to enable mineralization of a pollutant when metabolic cross-talk among different species is necessary, and is, therefore, carried out by bacterial consortia rather than by individual species (Bedard et al., 2006; Supaphol et al., 2006; Thomas et al., 2012). Third, metagenomics will provide appropriate metagenomic databases that will offer a rich stock of genes for the construction of novel microbial strains for targeted use in bioremediation efforts. Microbiologists consider the metagenomics-based bioremediation approach to be one of the most important and potent tools for the eradication of pollutants from the environment (Mazaheri Assadi and Tabatabaee, 2010; Satpute et al., 2010; Chandran and Das, 2011).

## Metatranscriptomics and proteomics in bioremediation

Metagenomics is an enormously potent tool by which the genetic makeup of the microorganisms inhabiting any environment can be described. However, metagenomics offers limited functions in elucidating gene expression and activity. The rapid development of metatranscriptomics (Poretsky and Ann Moran, 2011) and metaproteomics (Verberkmoes et al., 2009b) has made it possible to predict the functional activities of the microbial consortia. Metatranscriptomic studies can be used to ascertain the activity of genes within a given environment. The expression of the functional genomes within environmental samples can be determined by metatranscriptomics. Metatranscriptomics is of great interest for research related to environmental remediation. Recently, transcriptomic studies have been applied to the tdfA gene (an herbicide degrading gene), and the gene was successfully quantified (Nicolaisen et al., 2008). Although a number of studies have been carried out regarding the discovery and diversity of the functional genes in environmental samples, few studies have thoroughly compared their findings with actual bioremediation rates calculated from real biodegradation events (Jørgensen, 2007; Winderl et al., 2007).

Environmental proteomics offers better results when combined with other “omic” approaches, such as transcriptomics and metabolomics. To date, protein profiling related to the treatment of contaminated environments has primarily used SDS–PAGE (1D) to characterize the microorganisms and the ecology that is involved in the bioremediation process. Since ecological studies primarily focus on the natural adaptation of the microbes to the environments they inhabit, proteomics has been applied in studies that have provided insights into the mechanisms of adaptation, particularly to thermophilic conditions. The proteins of hyperthermophilic microorganisms are of great significance, as they possess an improved conformational stability that allows the thermophiles to remain active at elevated temperatures. By making the use of this property, the molecular basis of protein folding and conformational stability can be predicted. Proteomic approaches have often been employed to gain a more complete understanding of the physiological responses of microbes to xenobiotics, temperature changes and other stressors (Lacerda and Reardon, 2009). Proteomics approaches are also helpful in analyzing the physiological changes that microbes undergo during bioremediation. Despite lacking metagenomic sequences, Wilmes and Bond (2004) were able to explore the microbial mediated phosphorus removal from contaminated water by utilizing a metaproteomic approach. Similarly, Lacerda et al. (2007) used 2D gel electrophoresis in combination with mass spectroscopy and de novo sequencing to identify more than one hundred proteins from microbial communities that were exposed to cadmium contaminated water. Advancements in proteomics technology have led to the identification of novel genes and proteins during the anaerobic degradation of ethyl-benzene and toluene, as demonstrated in a study by Ebn et al. (2005). During the anaerobic biodegradation of toluene, a number of genes and associated proteins are expressed. The proteomics approach has revealed novel pathways of aerobic and anaerobic biodegradation of toxins, and therefore it provides a basis for the identification of novel proteins. Regulated proteins are in various metabolic categories, including the general stress response, oxidative stress response, transcription regulation, transport molecules, and energy metabolism (Santos et al., 2004). Konopka and Wilkins (2012) utilized meta-transcriptomics and meta-proteomics to identify and characterize a *Geobacter* spp. in response to carbon biostimulation alterations. Doré et al. (2015) investigated fungal response under various environmental conditions and identified proteins and extracellular enzymes by making use of “omic” approaches, along with combined liquid chromatography and mass spectrometry techniques.

## Microbial metabolomics and fluxomics in bioremediation

Metabolomics, the study of the metabolite profiles of a cell within a given set of conditions, is one of the most recent entries to the “omics” family. In addition to genomics, transcriptomics and proteomics, cutting-edge research is now expanding toward the analysis of microbial cellular metabolites. Metagenomics has already solidified its significance by playing an important role in understanding the diversity and functional aspects of the microbial consortia. Application of metabolome-based approaches to the environmental samples has made it possible to develop models that can envisage microbial activities under different bioremediation strategies. Metabolomics allows us to better understand the dynamic operations of the microbial communities and their functional contributions to the environments in which they live. When a microbial cell is subjected to an environmental stressor it releases a number of primary and secondary metabolites. The metabolomics approach explores the functional roles of these low-molecular weight metabolites. Recently, a number of studies have made use of microbial metabolome analysis to investigate the biodegradation of anthropogenic pollutants. An example of this is the comparative metabolome analysis of *Sinorhizobium* sp. during phenanthrene degradation (Keum et al., 2008). In this study, the intracellular metabolomes were compared with those from the carbon sources, and the metabolite profiles (fatty acids, polyhydroxy alkanoates, and polar metabolites) were analyzed with an untargeted metabolome analysis. Studies such as these show the importance of metabolomic data in bioremediation research. Villas-Bôas and Bruheim, 2007 explored the role of metabolomics in bioremediation research and described various experimental and conceptual approaches that have been developed for metabolomics and should be applied at bioremediation studies. Tang et al. (2007) evaluated the fluxome profile of *Shewanella*, a marine bacterial species known to possess co-metabolic pathways for the biodegradation of toxic metals, halogenated organic compounds and radionuclides. The analysis was carried out using biochemical, GC-MS, and statistical and genetic algorithms, and their results showed that *Shewanella* sp. exhibits a comparatively flexible metabolism flux when subjected to various carbon sources. Durand et al. (2010) performed a metabolic analysis of *Bacillus* sp. to define the metabolic pathways of this bacterial strain during the degradation of the herbicide mesotrione. Their analysis, which used liquid chromatography NMR and *ex situ* NMR, identified a total of six metabolites, of which the structures of four metabolites were suggested. Wharfe et al. (2010) applied FT-IR to monitor the biochemical and phenotypic changes in bacterial communities that could degrade aromatic compounds (i.e., phenol) that had been released from industrial bioreactors.

## Metagenomics in plant-microbe interactions and its significance for bioremediation

**Figure 4:**
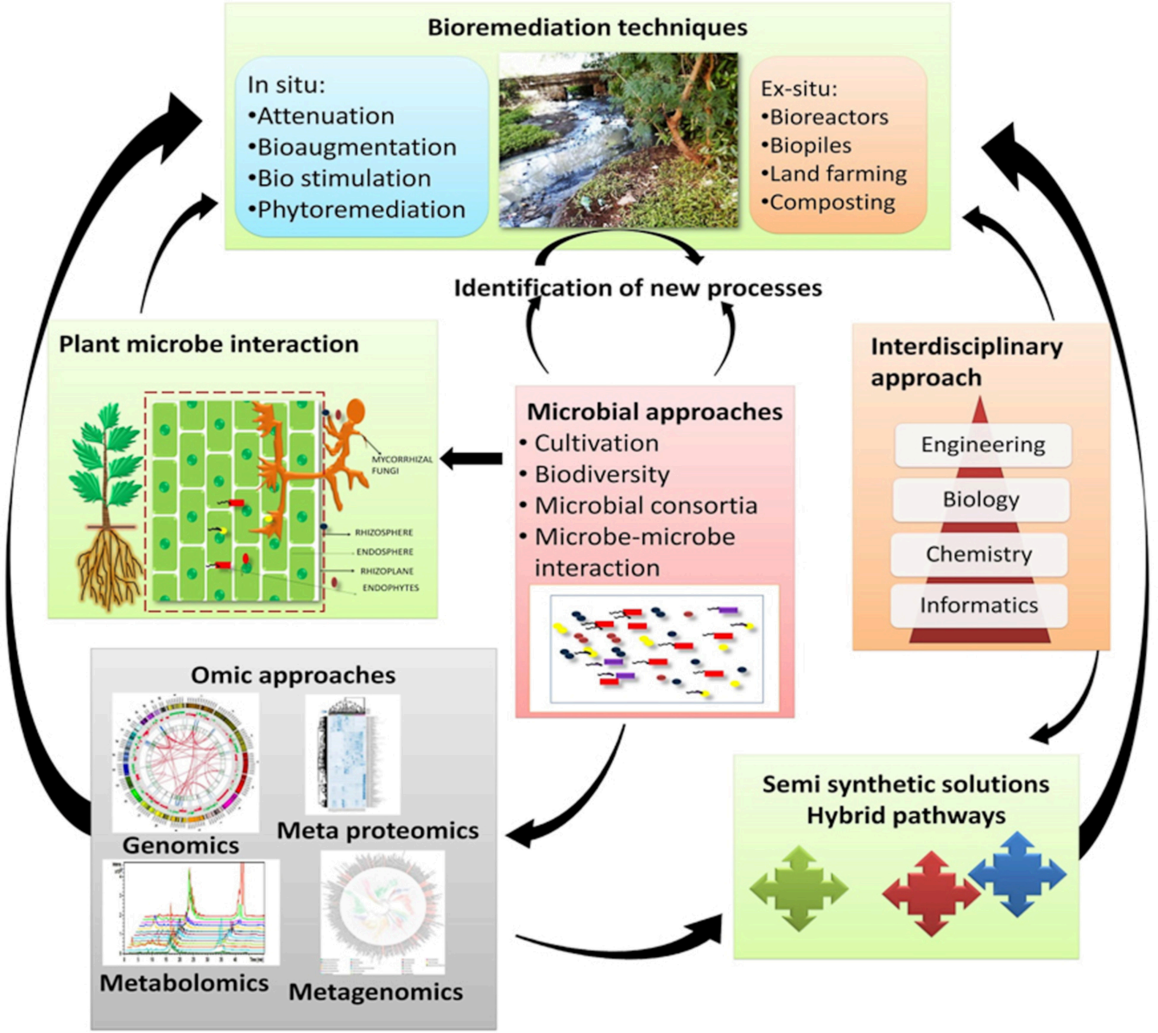
Novel processes in order to improve bioremediation of polluted environments.

Plant-omics is one of the fastest growing fields of science, due to the urgent need to address important questions faced by humanity respecting environmental remediation, ecological sustainability, medicine, biofuels, and agriculture (Gemperline et al., 2016). Although the plant-microbe interaction has been known for many years, its impact on the plant's hardiness and production was clarified only recently. Plant-microbe associations are extremely diverse in nature, with microbes prospering and thriving within the plant structures, both underground and above ground (Vorholt, 2012; Bulgarelli et al., 2015). Environmental pollution has increased over the past two decades as a result of both natural events and anthropogenic activities (Vácha et al., 2015). The widespread contamination of ecosystems resulting from metallurgical and mining activities, and atmospheric deposition from fossil fuel power plants has led to the accumulation of harmful toxic chemicals in the environment, such as PAHs. Long-term exposure to PAHs can cause damage to the respiratory system, central nervous system, and endocrine system, and can cause skin, liver, lung, bladder, and kidney cancers (Singh et al., 2004; Locksley, 2011). Therefore, the need to remediate PAH-contaminated environments is of great importance to protect both humans and animals. Plant-associated microbes can have phyllospheric, endophytic and rhizospheric interactions, and the interaction between these microbes and their host plants enables much of the plant growth and survivability outcomes in polluted environments. Plants can significantly improve bioremediation rates and outcomes, as they provide habitat and exchange nutrients with their microbial counterparts. In turn, the microbes improve the growth of plants by reducing the toxicity of soils via the removal of contaminants. Genome-enabled techniques offer a framework of plant-microbe interactions during environmental remediation. Metagenomic studies provide a way to unravel the plant-associated microbial diversity of the contaminated environments. This knowledge will form a basis for understanding indigenous microbial communities and will help to devise strategies for the remediation of contaminated environments (Figure 4).

## Standardization in bioremediation to overcome obstacles and to increase and enhance predictability

Globally, there are a number of environmental bioremediation sites that are undergoing *in situ* or *ex situ* bioremediation. Among these, many well-known cases involve the removal of both organic (e.g., explosives, TCE, solvents, dyes, etc.) and inorganic (e.g., nitrates, chlorides, uranium) pollutants (Nizzetto, 2010; He and McMahon, 2011). Many of these operations are not only examples for the elimination of environmental pollutants; they also function as model systems to increase our understanding of the biology within an ecosystem, including microbes, under natural conditions. In addition to the remarkable successes that are being achieved in the laboratory and, in some cases, in the environment, there have also been several failures (de Lorenzo, 2009). This demonstrates that it is not always easy to transform lab-based research to the field and that novel and pioneering attempts are necessary to increase the predictability of bioremediation results. The use of biology alone will not entirely eliminate pollution. It must be understood that successful bioremediation also requires the consideration of engineering aspects. Efforts are needed to minimize the operational start-up time and to set up fast and reliable sensors to monitor conditions that can lead to improved microbial effectiveness, when required (van der Meer and Belkin, 2010; Ramos et al., 2018).

Over the past few decades, advances in bioremediation have been largely achieved from culturable microbes that are easily obtainable and possess well-defined catalytic activities. However, much of the catabolic potential in nature remains unexplored, in part because microbiologists have not been able to replicate the very specific fundamental features (pH, nutrients, temperature, osmotic conditions, etc.) of their environment. Currently, “omics” technologies are being exploited to discover the hidden microbial potential prior to their cultivation, with the intent of rescuing enzymatic activities related to the degradation of environmental contaminants (Figure 4). Metagenomic technology has changed the way in which environmental microbiologists think, and intensive genome mining will surely allow a greater exploration of microbes that have biodegradation properties.

## *In silco* analysis in bioremediation

The metagenomic approaches described above have greatly increased our understanding of the physiological capabilities of microbes. However, to predict the functioning of microorganisms within an environment, a more holistic view of metabolism is required to illustrate the outcome of the thousands of individual reactions occurring simultaneously in a microbial cell. These descriptions are becoming possible due to advances in the development of *in silico* analyses (Cardoso et al., 2015). Bioinformatics, which has already made novel discoveries in the field of microbial ecology, is now expected to hasten the discovery of unexamined areas of the microbial universe. The *data deluge* has made bioinformatics an indispensable tool in modern day research; recent innovative technologies are generating a large amount of data at an unprecedented pace. The huge amount of data generated as a result of sequencing has placed high demands and burdens on computers and computational scientists. Bioinformatics relies on genomics and proteomics, and it holds great promise as a tool to address long-standing questions regarding the molecular mechanisms involved in biodegradation pathways (Fulekar, 2010). Bioinformatics has shown its novel capabilities in the field of bioremediation Bioinformatics has shown its novel capabilities in the field of bioremediation, however the resources that are devoted toward the bioremediational processes are still scarce. A number of novel and interesting projects have been carried with the aim to organize the bulk of data generated via multi-omics approaches. One prime example of such projects is “The University of Minnesota Biocatalysis/Biodegradation Database (UMBBD)” (Gao et al., 2010). Another example is “The Environmental Contaminant Biotransformation Pathway” (The enviPath tool) and was very recently launched as the updated version of EAWAGBBD/ PPS (Wicker et al., 2016). Some other prominent examples are those BioCyc and MetaCyc databases by SRI International (Caspi et al., 2016).

## Success stories: omics-approaches in microbial bioremediation

Microbial-bioremediation is generally viewed as a sustainable and cost effective technology, as it depends on microorganisms to transform the contaminants into benign compounds. Multi-omics techniques are capable of revolutionizing the biological treatment of contaminated environments by allowing highly sensitive characterization and functioning of the yet-uncultured microorganisms. To elucidate how, different omic-approaches can be applied to microbial-mediated bioremediation applications, here in this section, we have tried to mention some of the success stories of different “omic” approaches in microbial bioremediation.

Environmental biologists deem microbial bioremediation to be one of the potent tools to eliminate environmental contaminants (Bell et al., 2014; Chemerys et al., 2014; Roling, 2015). A number of review articles have recently been published and provide a much detailed account about the role of multi-omics (metagenomics, proteomics, metatranscriptomics, metabolomics, and fluxomics) approaches in microbial bioremediation. Recently “Omics”-based techniques revitalized the study of polycyclic aromatic hydrocarbons (PAH) catabolism by offering an integrative view regarding the biochemical processes responsible for the degradation of PAH in contaminated sites (El Amrani et al., 2015). In the past few years, a number of detailed review articles have been published based on various aspects of metagenomic approach and have highlighted its pledge for bioremediation (Sar and Islam, 2012; Bell et al., 2015; Nousiainen, 2015; Jung et al., 2016; Techtmann and Hazen, 2016; Duarte et al., 2017; Tripathi et al., 2018). Metagenomics indeed allowed the study of microbial communities within their whole complexity, including interactions among the community members. Since the complete mineralization of any pollutant requires metabolic-crosstalk between microbial communities and is hence performed by microbial consortia rather than individual species. In contrast to other bioremediational approaches (physical and chemical), metagenomic bioremediation provides the best results with better degrading ratios Recently a number of the studies have been carried that provide an insight of how proteomics help in bioremediation (Kim et al., 2003, 2004, 2007; Zhao et al., 2005). Similarly (Jennings et al., 2009) while using the integrative omic-approach of Proteomic and Transcriptomic revealed the genes that are up regulated by cisdichloroethene (cDCE) a suspected carcinogen in JS666 strain of *Polaromonas sp*. In another study (Holmes et al., 2009), while using the whole-genome microarray analysis decoded the transcriptome of *Geobacter uraniireducens* strain capable of growing in uranium-contaminated subsurface sediments. Many other prominent studies that discuss the importance and breakthroughs of the metatranscriptomics and proteomics in the field of microbial-mediated bioremediation are those carried out (Singh, 2006; Lee et al., 2012; Shukla, 2017; Niu et al., 2018). Now a days, it is a well-established fact that metabolome analysis serves as a potent approach for discovering novel metabolic pathways and networks (Weckwerth and Fiehn, 2002; Villas-Bôas et al., 2007). Metabolomics approach aims to quantify the functional role of the metabolites within the microbial cells via separation and analytical techniques, while as fluxomics aims at determining the metabolic fluxes. Bargiela et al. (2015), applied metabolomics techniques to three different chronically hydrocarbon (petroleum) polluted sites and revealed the importance of the general aerobic processes that were uncoupled with degradation. The results from their study showed the presence of more than 4,776 metabolite in these polluted sites, thereby revealing the high metabolic heterogeneity within the study sites. A number of other studies that have used metabolomic and fluxomics approaches to study the biodegradation of anthropogenic environmental pollutants were carried out by Villas-Bôas and Bruheim (2007), Wiechert et al. (2007), Keum et al. (2008), Tang et al. (2009), Brune and Bayer (2012).

## Systems biology approaches to bioremediation

Nature has its ways of cleaning the environments by eliminating the contaminants in order to maintain a perfect balance; however in this modern era of industrialization the rate at which the pollutants are released into the environment has crossed the threshold limit of the nature. Recently, the modern approaches such as genomics, transcriptomics, proteomics, metabolomics, and fluxomics have been applied to the systems biology of microbial consortia with in diverse array environments. Systems biology, an integrated research approach is used to study the intricate biological systems by exploring interactions and networks at different structural levels (molecular, cellular, community, and ecosystem). Integration of the results from various “omics-approaches” has offered crucial insights into the survival, metabolism and interaction of microbial communities within their native environments (Baker and Banfield, 2003; Hemme et al., 2010; Hu et al., 2010). Systems biology approaches are continuously being adopted to unravel key processes in order to understand, predict, optimize and appraise the survival strategies, and microbial function within the ecosystems of interest. However, for this approach to be successful, it needs to overcome some challenges, including the materials and reagents, amount of samples, high cost of sample processing, technocrats to process the samples, and data synthesis (Chakraborty et al., 2012). In order to gain an understanding of intricate *in situ* bioremediation processes, monitoring techniques, enzyme probes, genomics, transcriptomics, proteomics, metabolomics, and metabolomics provide unique insights into the important microbial reactions. Microbes, are able to directly immobilize and detoxify toxicants (Elias et al., 2003). As discussed, the multi-omics approaches have been major breach in providing much deeper insights both in the cellular function and gene products interacting within the environment (VerBerkmoes et al., 2009a). Immunomagnetic separations, a specific, efficient, rapid, and technically simple technique has been applied for the separation of the target microbes directly from microbial consortium (Chakraborty et al., 2011). The technique holds a great pledge in enabling the omic-based (proteomics, transcriptomics, metabolomics) studies directly on cells collected from the environment. Integrating all these techniques along with *insilco* analysis and modeling will enable novel break-through in the field of environmental biotechnology. Since 2004, a number of research groups all over the world have been involved in the active implementation of the basic to understand and comprehend the systems biology of contaminated environments and predicting feasible and practicable remediation technologies. Systems biology approaches have successfully been applied to various environmental contaminants, some of the notable reviews on systems biology approaches for radionuclides are (Palumbo et al., 2004; Fields et al., 2006; Cardenas et al., 2008; Conrad et al., 2010), for hydrocarbons (Harayama et al., 2004; de Lorenzo, 2008; Fredrickson et al., 2008; Atlas and Hazen, 2011; Zhou et al., 2011), for metals (Fredrickson et al., 2008; Hubbard et al., 2008; Han et al., 2010; Liu et al., 2011), and for chlorinated solvents (Song et al., 2002; Lehman et al., 2004; Erwin et al., 2005; Scow and Hicks, 2005; Rahm et al., 2006; Cupples, 2008; Illman and Alvarez, 2009; Werner et al., 2009; Conrad et al., 2010).

## Future perspectives

Considering the present state of “omics,” future research in microbe-based bioremediation may focus on the following:

1. Mining of the current data to provide additional insights into bioremediation pathways (mechanisms).
2. Numerical modeling and simulation of the data, which requires the advancement of novel algorithms.
3. Standardization of protocols for the collection, analysis, reposition, and transmission of data.
4. Identification and characterization of novel indicators (biomarkers). These indicators may help to determine the use of particular bioremediation operations.
5. Integration of the data generated via a number of the “omics” approaches. By integrating metagenomics (functional), transcriptomics, proteomics and metabolomic data, researchers may have a more clear and complete understanding of microbe-based bioremediation pathways and could provide a thorough and detailed perspective on the microbial consortia needed for the bioremediation process.

## Concluding remarks

Considering the immense threat posed by widespread environmental contamination by xenobiotics and other toxic chemicals, novel methods for decontamination, and clean-up are urgently required. Since, the interaction between the microbial communities and the environmental contaminants are far from being simple, and it is challenging to understand and explore the extreme environments, these microorganisms inhabit. Environmental contamination can be viewed as an ecological malaise and for such a malaise; bioremediation can be prescribed as a “perfect medicine.” Toward a much deeper perceptive and understanding of the microbial-mediated bioremediation, novel omic-approaches (genomics, transcriptomics, proteomics, metabolomics, and fluxomics) presents a remarkable pledge as tools to study the mechanisms involved in various bioremediational pathways. The integrative approach of these techniques in this era of “omics” has paved way for the successful execution of the efficient bioremediational strategies. The applications of these approaches are still in their infancy; but the bulk of data that is constantly being generated by the present day omic technocrats needs to be organized within the informative databases. Omics-approaches show great ability to predict organism's metabolism in polluted environments and to envisage the microbial-mediated attenuation of the pollutants to fasten bioremediation. The study of molecular mechanisms behind the microbial transformations of the toxic pollutants using omic-approaches to bioremediation would aid in tracking the responsible organisms and in efficient elimination of the contaminants from the environments.

## Author contributions

MM prepared the draft of manuscript under the guidance of AK and SY. AD prepared the illustrations. EA and AH edited the manuscript.

### Conflict of interest statement

The authors declare that the research was conducted in the absence of any commercial or financial relationships that could be construed as a potential conflict of interest.
