# Supplementary material for: Understanding and Designing the Strategies for the Microbe-Mediated Remediation of Environmental Contaminants Using Omics Approaches
Source: Front Microbiol. 2018 Jun 4;9:1132. doi: 10.3389/fmicb.2018.01132 (PMC5994547; doi:10.3389/fmicb.2018.01132)
Supplement: Supplementary file 1 [file Table_1.docx]

Supplementary Table S1: Common terminologies

| Terminologies | Definition |
| --- | --- |
| Microbiome | Sum total of all the microorganisms populating a particular environment. For example, gut microbiome may refer to all microbes residing within a specific intestinal tract; however the composition of microbiome may vary significantly among the individuals |
| Bioremediation | Refers to the application of biological agents, *viz.,* fungi, bacteria and green plants in order to eliminate or counterbalance the contaminants in polluted environments. Bacteria and fungi usually function by breaking down the pollutants such as xenobiotics, petroleum etc into less harmful substances |
| Next generation sequencing or High throughput technologies | Next- generation sequencing or high-throughput sequencing, is the catch-all term that are used to illustrate a series of different modern sequencing technologies including; ion Torrent, Illumina, and Oxford Nanopore sequencing. |
| Genomics | An interdisciplinary field of biological science concerned with the application of molecular biology as well as genetics techniques to genetic mapping and DNA sequencing of either a set of genes or the whole genomes from selected organisms, by systematizing the results within the databases. |
| Metagenomics | It is defined as culture-independent genome-level identification and characterization of different microbial communities *via* high-throughput sequencing technologies, aimed at exploring the transorganismal behaviours of communities along with the environment they inhabit. |
| Proteomics | It is defined as the large-scale experimental study of proteomes of an organism, tissue or cell within a well defined set of conditions. The entire set of the proteins produced by an organism is proteome. |
| Metabolomics | It refers to the study of chemical processes involving metabolites Metabolomics provide immediate information regarding the metabolites with in a biological sample (cell, tissue or organism). Unlike other “omics” approaches metabolomics is most powerful as it directly reflects the biochemical activities as well as the state of the cells or tissues. |
| Metatranscriptomics | It refers to the study of activity as well as function of the entire RNA-sequences (transcripts) obtained directly from the environmental samples. |
| Fluxomics | It refers to the techniques that seek to determine the metabolic fluxes which in turn determine the cellular phenotypes. It falls in the domain of systems biology which is evolved through the emergence of high throughput technologies. |
